# Mortality and causes of death in patients with atrial fibrillation: A nationwide population-based study

**DOI:** 10.1371/journal.pone.0209687

**Published:** 2018-12-26

**Authors:** Euijae Lee, Eue-Keun Choi, Kyung-Do Han, HyunJung Lee, Won-Seok Choe, So-Ryoung Lee, Myung-Jin Cha, Woo-Hyun Lim, Yong-Jin Kim, Seil Oh

**Affiliations:** 1 Department of Internal Medicine, Seoul National University Hospital, Seoul, Republic of Korea; 2 Department of Biostatistics, College of Medicine, The Catholic University of Korea, Seoul, Republic of Korea; 3 Department of Internal Medicine, Sejong General Hospital, Bucheon, Republic of Korea; 4 Department of Internal Medicine, SoonChunHyang University Hospital Seoul, Seoul, Republic of Korea; 5 Department of Internal Medicine, Seoul National University Seoul Metropolitan Government Boramae Medical Center, Seoul, Republic of Korea; University of Palermo, ITALY

## Abstract

**Background:**

Patients with atrial fibrillation are known to have a high risk of mortality. There is a paucity of population-based studies about the impact of atrial fibrillation on the mortality risk stratified by age, sex, and detailed causes of death.

**Methods:**

A total of 15,411 patients with atrial fibrillation from the Korean National Health Insurance Service-National Sample Cohort were enrolled, and causes of death were identified according to codes of the 10th revision of the International Classification of Diseases.

**Results:**

From 2002 to 2013, a total of 4,479 (29%) deaths were confirmed, and the crude mortality rate for all-cause death was 63.3 per 1,000 patient-years. Patients with atrial fibrillation had a 3.7-fold increased risk of all-cause death compared with the general population. The standardized mortality ratio for all-cause death was the highest in young patients and decreased with increasing age (standardized mortality ratio 21.93, 95% confidence interval 7.60–26.26 in patients aged <20 years; standardized mortality ratio 2.77, 95% confidence interval 2.63–2.91 in patients aged ≥80 years). Women with atrial fibrillation exhibited a greater excess mortality risk than men (standardized mortality ratio 3.81, 95% confidence interval 3.65–3.98 in women; standardized mortality ratio 3.35, 95% confidence interval 3.21–3.48 in men). Cardiovascular disease was the leading cause of death (38.5%), and cerebral infarction was the most common specific disease. Patients with atrial fibrillation had an about 5 times increased risk of death due to cardiovascular disease compared with the general population.

**Conclusions:**

Patients with atrial fibrillation had a 4 times increased risk of mortality compared with the general population. However, the impact of atrial fibrillation on mortality decreased with age and in men. Cerebral infarction was the most common cause of death, and more attention should be paid to reducing the risk of stroke.

## Introduction

Atrial fibrillation (AF) is the most common arrhythmia, with gradually increasing incidence and prevalence worldwide [1, 2]. AF is associated with an increased risk of mortality after adjusting for cardiovascular comorbidities [3]. Previous studies have reported that AF is closely associated with an increased risk of all-cause and cardiovascular mortality [4–6]. However, most previous studies that investigated the prognosis of AF were performed in Western populations [4–8], and few studies have reported mortality rates associated with AF in Asians [9–11]. In a prospective registry of 46 countries across 8 geographical regions, the 1-year mortality rate in South America and Africa was twice that in North America, Western Europe, and Australia, suggesting an inter-regional and ethnic difference in mortality in patients with AF [9]. Considering the differences between Asians and Westerners in terms of life expectancy, prevalence of cardiometabolic risk factors, and availability and types of medical and social welfare systems, the prognostic significance of AF on mortality understandably differs between the 2 populations.

A recent meta-analysis evaluated the differential prognostic effects of AF based on sex, and indicated that AF was significantly associated with a higher risk of all-cause and cardiovascular mortality in women than in men [4]. However, whether the effect of AF on mortality varies with age is unclear. Additionally, no population-based study has thoroughly investigated the cause of death in patients with AF. Therefore, in this study, we investigated the mortality patterns and closely analyzed the cause of death in patients with AF by using a nationwide population-based cohort.

## Materials and methods

### Database

The National Health Insurance Program in Korea operated by the National Health Insurance Service (NHIS) is a mandatory medical care system for the Korean population (approximately 50 million people). The NHIS stores and manages an accurate database of healthcare practices and prescriptions in Korea. The NHIS-National Sample Cohort (NHIS-NSC) is a population-based cohort created by the NHIS. The NHIS-NSC provides medical researchers with representative information about the healthcare system utilization and health examinations of Korean citizens. The cohort comprises 1,025,340 individuals representing 2.2% of the entire Korean population in 2002. The subjects were selected through systematic stratified random sampling with 1,476 strata that consisted of 18 groups according to age, 2 groups according to sex, and 41 groups according to income level. The representativeness of the cohort was assessed by examining whether the 95% confidence interval (CI) of the average total annual medical expense of the sample represents the population average. In addition, the cohort was compared to the general population according to residence distribution across 16 regions in Korea. Detailed information about the NHIS-NSC has been described elsewhere [12, 13]. We received approval to use the database released by the NHIS in 2014, which included patients observed between 2002 and 2013.

### Study population

According to the revised 10th International Statistical Classification of Diseases and related health problems (ICD-10) codes, AF was defined as I48 to include patients requiring ≥2 outpatient visits or ≥1 hospitalization per year. To ensure that the study included only patients with nonvalvular AF, we excluded those with mitral stenosis (I50, I52, and I59) or preexisting mechanical heart valves (Z952–Z945). The codes for comorbidities such as hypertension (I10–I15), diabetes mellitus (E11–E14), congestive heart failure (I50), stroke (I63 or I64), transient ischemic attack (TIA) (G458 or G459), thromboembolism (I74), myocardial infarction (I21 or I22), and/or peripheral artery disease (I70 or I73) are summarized in S1 Table, and validated in previous studies [2, 14–17]. The CHA_2_DS_2_-VASc (congestive heart failure/left ventricular dysfunction, hypertension, age ≥75 years, diabetes mellitus, previous stroke or TIA or thromboembolism, vascular disease, age 65–74 years, female sex) score was calculated for each patient by assigning 1 point each for age between 65 and 74 years, female sex, and the presence of hypertension, diabetes mellitus, heart failure, and vascular disease (prior myocardial infarction or peripheral artery disease), and adding 2 points each for a history of stroke/TIA/thromboembolism or age ≥75 years [18].

Patients were stratified into 10-year age groups; young (<20) and old (>80) patients were grouped together because of their relatively small numbers. All patients underwent follow-up from the start date of the study to the end of 2013 or the day of disqualification because of death or emigration.

### Mortality and causes of death

We used the mortality database provided by Statistics Korea, a government agency. This mortality database includes death-related data such as age at the time of death, as well as the causes, date, and place of death. The causes of death were coded based on the Korean Standard Classification of Diseases and Causes of Death, which is based on the ICD-10 system. The 2 datasets were matched using the resident registration number, which is a unique personal identification system used in Korea. Finally, all provided data were de-identified.

### Statistical analysis

The number of deaths and person-years of follow-up were analyzed, and mortality rates were calculated based on the number of deaths per 1,000 person-years. The standardized mortality ratio (SMR) was used to compare the mortality rate with that in the general population. SMR is the ratio of the observed deaths in the population with AF to the expected deaths based on data from the general Korean population. The expected mortality was calculated using the mortality rate of the mean of the midyear population between 2002 and 2013 from the Korean Statistical Information Service (Statistics Korea, Causes of Death Statistics, 2002–2013). The causes of death, cause-specific mortality rates, and cause-specific SMRs were computed based on ICD-10 codes. SMR and 95% CI were calculated using Byar’s approximation [19]. Survival analysis of patients with AF based on the CHA_2_DS_2_-VASc score was performed using the Kaplan-Meier method. All statistical analyses were performed using SAS software version 9.4 (SAS Institute Inc., Cary, NC, USA).

### Ethical statement

This study was approved by the NHIS Review Committee (2017-2-287) and was exempted from review by the Seoul National University Hospital Institutional Review Board (1612-037-812). The subjects were not identified because the database was de-identified and anonymized from the beginning. Therefore, informed consent could not be obtained.

## Results

### Patient characteristics

Among the 1,025,340 individuals whose information was available in the NHIS-NSC database, we identified 15,411 patients with AF between 2002 and 2013. The baseline characteristics of patients with AF are shown in S2 Table. The mean patient age was 63.9 ± 15.9 years, and 8,226 (53.4%) of the patients were men. Two-thirds of patients with AF were aged ≥60 years (n = 10,292, 66.7%) and 16.9% of patients were aged <50 years (n = 1,232). The mean CHA_2_DS_2_-VASc score was 2.9 ± 1.9. At the time of identification, we found 5,363 (34.8%), 1,353 (8.8%), and 3,674 (23.8%) patients with ischemic heart disease, myocardial infarction, and congestive heart failure, respectively. A previous stroke or TIA was noted in 2,384 (15.5%) patients. Approximately 18% of patients with AF received oral anticoagulants (OACs), and 11% of patients received antiarrhythmic drugs (AADs) in the same year as the AF diagnosis. Patients with OAC use were older (65.3 ± 12.7 vs. 63.6 ± 16.6 years, p < 0.001) and had higher mean CHA_2_DS_2_-VASc scores (3.5 ± 1.9 vs. 2.8 ± 2.0, p < 0.001) than patients without OAC use. Patients with AAD use had more OAC therapies than those without AAD use (32.1% vs. 16.1%, p < 0.001).

### All-cause mortality in patients with atrial fibrillation

**Fig 1:**
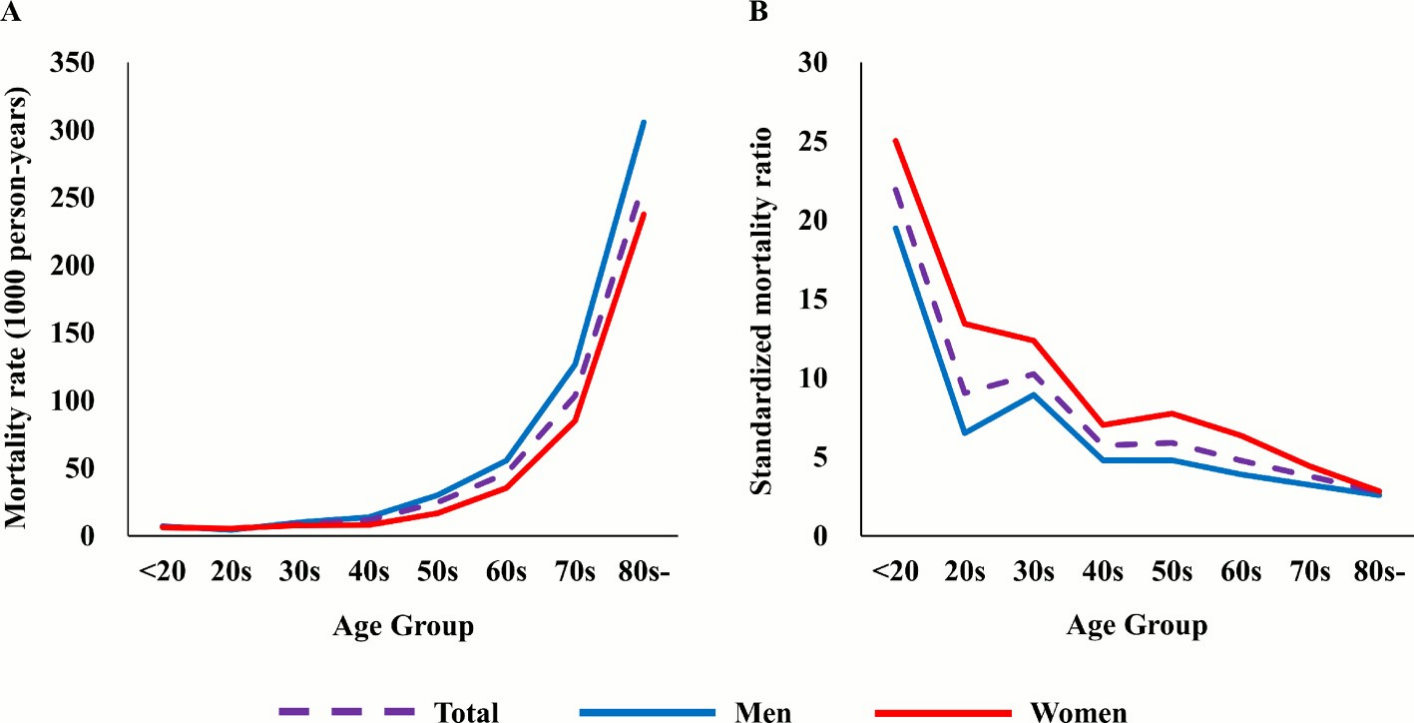
Mortality rates and standardized mortality ratios (SMR) of patients with atrial fibrillation (AF) according to age and sex. (A) The mortality rates of patients with AF increased with age. Male patients with AF showed higher mortality rates than female patients with AF. (B) The SMR of patients with AF decreased with age. Female patients with AF showed higher SMR than male patients with AF.

We observed 4,479 deaths during a total of 70,791 person-years of follow-up. The age- and sex-specific mortality rate and SMR for all-cause death are presented in Table 1 and Fig 1. Overall, in patients with AF, the crude mortality rate for all-cause death was 63.3 per 1,000 person-years. Patients with AF demonstrated a 3.67-fold higher risk of all-cause death than an age- and sex-matched general population (SMR 3.67, 95% CI 3.56–3.78).

**Table 1:** The age- and sex- specific all-cause mortality rate and standardized mortality ratio of patients with atrial fibrillation.

	Total					Men				Women			
	N	Death	P-Y	MR^a^	SMR (95% CI)	Death	P-Y	MR	SMR (95% CI)	Death	P-Y	MR	SMR (95% CI)
**<20**	215	9	1,321	6.8	21.93 (7.60–36.26)	6	841	7.1	19.50 (3.90–35.10)	3	479	6.3	25.02 (0.00–53.34)
**20s**	348	11	2,240	4.9	9.04 (3.70–14.39)	5	1,159	4.3	6.50 (0.80–12.20)	6	1,081	5.5	13.44 (2.69–24.20)
**30s**	669	36	4,018	9.0	10.26 (6.91–13.61)	22	2,225	9.9	8.93 (5.20–12.67)	14	1,792	7.8	12.37 (5.89–18.86)
**40s**	1,372	95	8,223	11.6	5.73 (4.58–6.88)	70	5,101	13.7	4.79 (3.67–5.91)	25	3,121	8.0	7.02 (4.27–9.78)
**50s**	2,515	333	13,438	24.8	5.89 (5.26–6.52)	246	8,180	30.1	4.80 (4.20–5.39)	87	5,257	16.5	7.74 (6.12–9.37)
**60s**	3,875	949	20,460	46.4	4.77 (4.47–5.08)	618	11,096	55.7	3.90 (3.59–4.21)	331	9,363	35.4	6.35 (5.66–7.03)
**70s**	4,118	1,618	15,624	103.6	3.78 (3.59–3.96)	883	6,972	126.6	3.22 (3.01–3.43)	735	8,652	85.0	4.40 (4.08–4.72)
**≥80**	2,299	1,428	5,468	261.1	2.77 (2.63–2.91)	577	1,887	305.7	2.58 (2.37–2.79)	851	3,581	237.6	2.81 (2.62–3.00)
**Overall**	15,411	4,479	70,791	63.3	3.67 (3.56–3.78)	2,427	37,462	64.8	3.35 (3.21–3.48)	2,052	33,329	61.6	3.81 (3.65–3.98)

^a^MR was presented as the number of deaths per 1,000 person-year.

AF, atrial fibrillation; CI, confidence interval; MR, mortality rate; P-Y, person-year; SMR, standardized mortality ratio.

**Fig 2:**
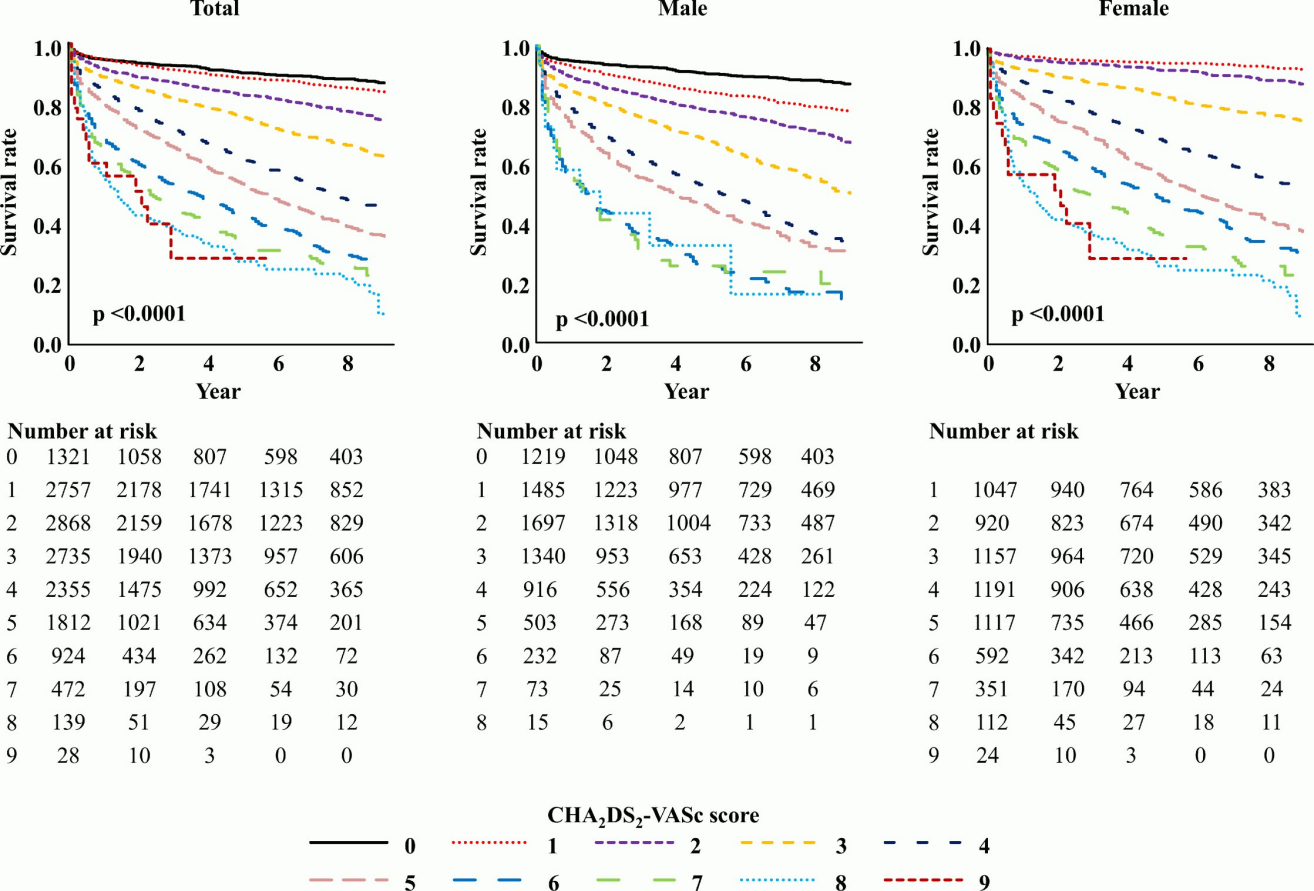
Kaplan-Meier survival curves according to CHA_2_DS_2_-VASc score. Survival rate was lower in atrial fibrillation patients with higher CHA_2_DS_2_-VASc score.

The crude mortality rate was higher in men than in women (64.8 vs. 61.6 per 1,000 person-years). We investigated the sex-specific SMR to determine the prognostic significance of AF based on sex. Women showed a higher SMR than men. Women with AF showed a 3.81-fold higher mortality risk than women from the general population (SMR 3.81, 95% CI 3.65–3.98); however, men showed a 3.35-fold increased risk (SMR 3.35, 95% CI 3.21–3.48). The age-specific all-cause mortality rate increased with age, particularly beyond 60 years (from 4.9 per 1,000 person-years in those in the 20s to 261.1 per 1,000 person-years in those aged ≥80 years). With respect to age-specific SMR, the value was the highest in the younger age groups and decreased with increasing age (SMR 21.93, 95% CI 7.60–26.26 in those aged <20 years vs. SMR 2.77, 95% CI 2.63–2.91 in those aged ≥80 years; Table 1). The all-cause SMRs were higher in women than in men across all age groups. The mortality rate in patients with OAC use was lower than that in patients without OAC use, although patients with OAC use were older and had higher CHA_2_DS_2_-VASc scores (23.0% vs. 30.4%, p < 0.001). The mortality rate in patients with AAD use was lower than that in patients without AAD use (14.5% vs. 31.0%, p < 0.001). The CHA_2_DS_2_-VASc score showed a close correlation with overall survival in both men and women (Fig 2).

### Cause of death in patients with atrial fibrillation

The causes of death observed in patients with AF differed from those in the entire NHIS-NSC cohort (Table 2). In patients with AF, diseases of the circulatory system (38.0%) were the most common cause of death, followed by malignant neoplasms (23.4%) and diseases of the respiratory system (8.4%). The most common cause of death in the entire cohort was malignant neoplasms (27.6%), followed by diseases of the circulatory system (22.4%) and injury, poisoning, and other external causes (12.1%) (Table 2 and S1 Fig).

**Table 2:** Major causes of death in entire cohort and in patients with atrial fibrillation according to ICD-10 codes.

Rank	Total Cohort		AF patients	
	ICD-10 codes	Number (%)	ICD-10 codes	Number (%)
**1**	Malignant neoplasms (C)	15,440 (27.6)	Diseases of the circulatory system (I)	1,701 (38.0)
**2**	Diseases of the circulatory system (I)	12,546 (22.4)	Malignant neoplasms (C)	1,046 (23.4)
**3**	Injury, poisoning and certain other consequences of external causes (S & T)	6,779 (12.1)	Diseases of the respiratory system (J)	372 (8.4)
**4**	Symptoms, signs and abnormal clinical and laboratory findings, not elsewhere classified (R)	5,978 (10.7)	Symptoms, signs and abnormal clinical and laboratory findings, not elsewhere classified (R)	275 (6.2)
**5**	Diseases of the respiratory system (J)	3,669 (6.6)	Endocrine, nutritional and metabolic diseases (E)	253 (5.7)
**6**	Endocrine, nutritional and metabolic diseases (E)	2,649 (4.7)	Injury, poisoning and certain other consequences of external causes (S & T)	195 (4.4)
**7**	Diseases of the digestive system (K)	2,496 (4.5)	Diseases of the digestive system (K)	156 (3.5)
**8**	Certain infectious and parasitic diseases (A & B)	1,332 (2.3)	Diseases of the genitourinary system (N)	124 (2.8)
**9**	Diseases of the nervous system (G)	1,303 (2.3)	Certain infectious and parasitic diseases (A & B)	108 (2.4)
**10**	Mental, behavioral and neurodevelopmental disorders (F)	1,025 (1.8)	Diseases of the nervous system (G)	71 (1.6)

AF, atrial fibrillation; ICD, international statistical classification of diseases and related health problems.

**Fig 3:**
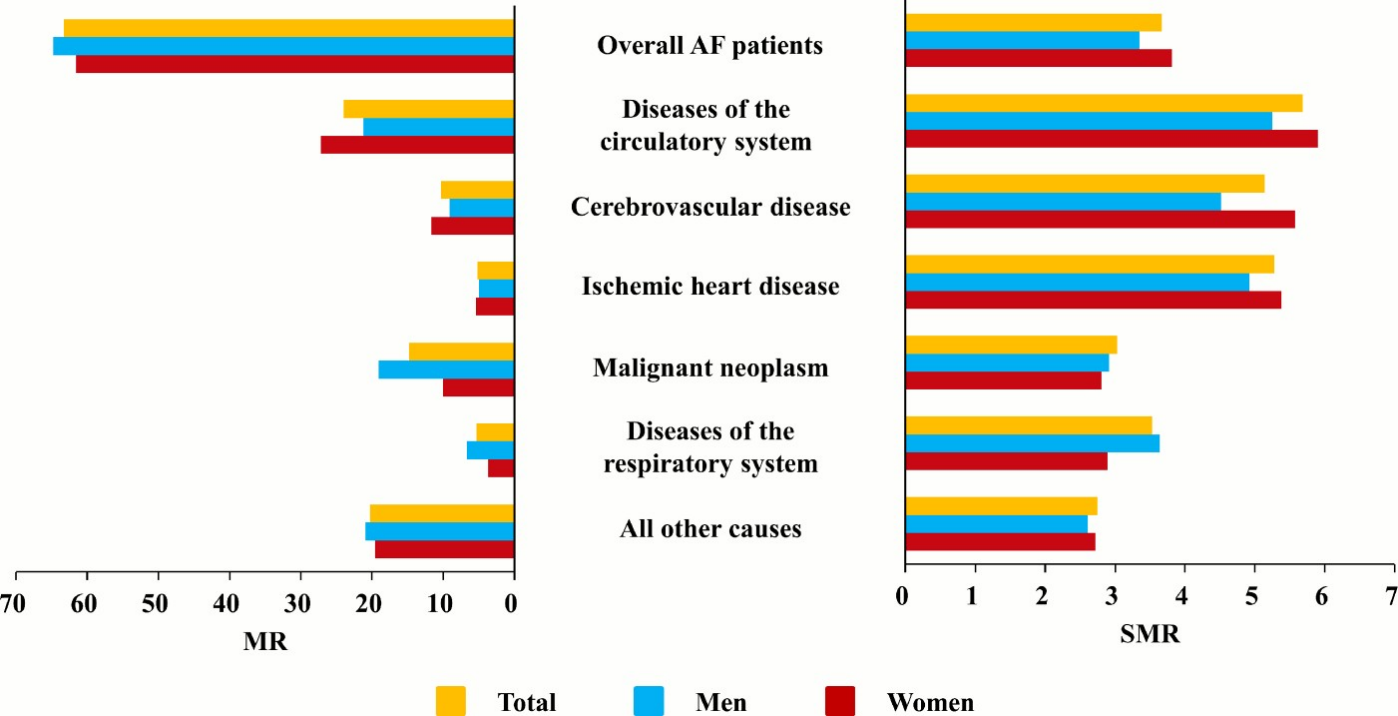
Mortality rates (MR) and standardized mortality ratios (SMR) of specific causes of death in atrial fibrillation patients according to ICD-10 codes. The overall mortality rate (MR) was higher in men than in women, but the MR due to diseases of the circulatory system was lower in men. The SMRs were generally higher in women than in men except for malignant neoplasms and diseases of the respiratory system.

With respect to specific disease entities, cerebral infarction (I63, 7.8%) was the most common cause of death in patients with AF, followed by lung cancer (C34, 6.0%), myocardial infarction (I21, 5.0%), senility (R54, 4.5%), and sequelae of cerebrovascular diseases (I69, 4.5%). The causes of death differed between women and men. Cerebral infarction (I63, 9.5%) was the most common cause of death in women with AF, followed by senility (R54, 6.6%), sequelae of cerebrovascular disease (I69, 5.3%), acute myocardial infarction (I21, 5.1%), and heart failure (I50, 4.2%), whereas lung cancer (C34, 8.9%) was the most common cause among men with AF, followed by cerebral infarction (I63, 6.3%), acute myocardial infarction (I21, 4.8%), malignant neoplasms of the liver and intrahepatic bile ducts (C22, 4.1%), and chronic obstructive pulmonary disease (J44, 3.7%) (S3 Table). In patients with AF aged <60 years, malignant neoplasms were the most common cause of death followed by cardiovascular diseases. However, this pattern was reversed in those aged ≥60 years (S2 Fig). The cause-specific SMR indicated that patients with AF showed a higher risk of death due to cardiovascular disease (SMR 5.69), malignancy (SMR 3.03), respiratory disease (SMR 5.30), and other causes (SMR 2.75) than the general population. The overall SMR and the SMRs for cardiovascular diseases were higher in women with AF than in men with AF, whereas the SMRs for malignancy and respiratory disease were higher in men with AF than in women with AF (Table 3 and Fig 3).

**Table 3:** The cause-specific mortality rate and standardized mortality ratio in patients with atrial fibrillation according to ICD-10 code (the first code).

	Total			Men			Women		
	Death^a^	MR^b^	SMR (95% CI)	Death	MR	SMR (95% CI)	Death	MR	SMR (95% CI)
**Diseases of the circulatory system (I)**	1,701 (38.0)	24.0	5.69 (5.42–5.96)	796 (32.8)	21.2	5.25 (4.89–5.61)	905 (44.1)	27.2	5.91 (5.52–6.29)
** Cerebrovascular diseases (I60-9)**	732 (16.3)	10.3	5.14 (4.77–5.51)	341 (14.1)	9.1	4.51 (4.04–4.99)	391 (19.1)	11.7	5.57 (5.02–6.13)
** Ischemic heart diseases (I20-5)**	368 (8.2)	5.2	5.28 (4.74–5.82)	189 (7.8)	5.0	4.92 (4.22–5.62)	179 (8.7)	5.4	5.38 (4.59–6.17)
** Hypertensive diseases (I10-3)**	151 (3.4)	2.13	5.47 (4.59–6.34)	63 (2.6)	1.68	6.6 (4.97–8.23)	88 (4.3)	2.64	4.94 (3.91–5.97)
** Heart failure (I50)**	145 (3.2)	2.04	6.99 (5.85–8.13)	59 (2.4)	1.57	7.85 (5.85–9.86)	86 (4.2)	2.58	6.56 (5.17–7.94)
** Other circulatory diseases**	305 (6.8)	4.31	7.91 (7.02–8.79)	144 (5.9)	3.84	6.99 (5.85–8.13)	161 (7.8)	4.83	8.51 (7.2–9.83)
**Malignant neoplasms (C)**	1,046 (23.4)	14.8	3.03 (2.84–3.21)	714 (29.4)	19.1	2.91 (2.70–3.13)	332 (16.2)	10.0	2.81 (2.51–3.11)
**Diseases of the respiratory system (J)**	372 (8.4)	5.3	5.30 (3.53–3.17)	250 (10.3)	6.7	3.64 (3.28–4.09)	122 (6.0)	3.7	2.89 (2.38–3.40)
**Other causes**	1,434 (32.0)	20.3	2.75 (2.61–2.89)	782 (32.2)	20.9	2.61 (2.43–2.79)	652 (31.8)	19.6	2.72 (2.51–2.92)
**Overall**	4,479 (100)	63.3	3.67 (3.56–3.78)	2,427 (100)	64.8	3.35 (3.21–3.48)	2,052 (100)	61.6	3.81 (3.65–3.98)

^a^Death events were presented as number (percentage).

^**b**^MR was presented as the number of death per 1,000 person-year.

AF, atrial fibrillation; CI, confidence interval; ICD, international statistical classification of diseases and related health problems; MR, mortality rate; SMR, standardized mortality ratio

### Mortality due to specific diseases of the circulatory system in patients with atrial fibrillation

Cerebrovascular disease (I60-69) was the most common cause (732 patients, 43.0%) among deaths due to diseases of the circulatory system (1,701 deaths). Notably, cerebral infarction (I63) was the most common specific disease, accounting for 47.1% of cerebrovascular diseases (S3 Table). Ischemic heart disease (I20-25) was the second most common specific cause of death, followed by hypertensive diseases and heart failure. The cause-specific SMR showed that patients with AF demonstrated a higher risk of death related to cerebrovascular disease (SMR 5.14), ischemic heart disease (SMR 5.28), hypertensive diseases (SMR 5.47), and heart failure (SMR 6.99). Women with AF showed a higher mortality rate and SMR related to cerebrovascular disease and ischemic heart disease than men with AF (Table 3).

## Discussion

In this population-based study, we observed that patients with AF had a 3.67-fold higher risk of all-cause death than an age- and sex-matched general population. Women with AF showed a higher SMR than men with AF, suggesting that the effect of AF on death was greater in women than in men. The leading cause of AF-associated deaths was diseases of the circulatory system, and cerebral infarction was the most common cause of death. Patients with AF showed a 5.5-fold higher risk of death due to diseases of the circulatory system than the general population.

In the current study, the crude mortality rate for all-cause death was 63.3 per 1,000 person-years, which was similar to the all-cause mortality rate of 60.5 per 1,000 person-years reported by the Framingham study [3]. A Japanese study reported a lower crude mortality rate of all-cause deaths (21.7 per 1,000 person-years); however, the study included only a small number of patients with AF (n = 60) [11]. We observed that patients with AF in Korea had a 3.7-fold higher risk of mortality than the general population, which is higher than that reported in Europe and the United States [5, 20–22]. In a Swedish study, the risk of mortality in patients with AF was 1.6-fold higher than that in the general population [5]. A Serbian population-based observational study showed that the risk of all-cause mortality was 2.43-fold higher and that of cardiovascular mortality was 3.03-fold higher in patients with AF than in the general population [20]. However, those studies included only a limited number of patients. In the United States, AF was associated with a 1.5- to 1.9-fold increased risk of death in both men and women [21], and a similar result was reported in another study [22]. We observed that patients with AF showed a higher risk of mortality than an age-matched general population. Age has been considered a strong risk factor for mortality and morbidity in patients with AF, although other causes of death are also known to play an important role in older individuals [23]. A higher prevalence of permanent or persistent AF and a higher CHA_2_DS_2_-VASc score may contribute to unfavorable outcomes in old age [24]. Therefore, the mortality risk in older patients with AF is higher than that in younger patients with AF. However, the impact of AF on mortality differs with age. In younger patients with AF, the absolute mortality (number of deaths) was lower, but the impact of AF on mortality was higher, than that in older patients. In contrast, the absolute mortality was higher in older patients with AF, although the SMR was observed to be lower than that in younger patients with AF, which suggests that the impact of AF on mortality weakens with aging. As individuals without AF become older, they develop concomitant comorbidities other than AF, which could contribute to an increased risk of mortality, thereby weakening the impact of AF in older patients with AF. A possible explanation for this observation could be the multifactorial etiologies of AF noted in younger patients. Congenital heart diseases are known to be an important etiological factor in young patients with AF, which could also affect survival [25]. Familial AF, which develops at a relatively young age, shows a strong relationship with genetic mutations and might affect the long-term prognosis [26]. An excessive lifestyle, including heavy consumption of alcohol and excessive endurance exercise, tends to predispose individuals to the development of AF compared with the general population, and could affect survival [27, 28]. To our knowledge, this is the first population-based study including an adequate number of patients (n = 2,604, aged <50 years) that reports an increased impact of AF on mortality particularly in young patients.

Female sex is an important risk factor for stroke in patients with AF; thus, it has been incorporated in the CHA_2_DS_2_-VASc score [23]. The effect of sex on the survival of patients with AF remains controversial. AF has been shown to be a stronger risk factor for stroke and cardiovascular death in women than in men [7, 22]. This tendency has also been observed in patients at a low risk [29]. However, a German study demonstrated a similar or comparable risk of stroke between women and men with AF [30]. Renoux et al. reported that the case fatality was higher in men than in women [31]. Two studies on Asians have also shown similar results [10, 32]. The Framingham study showed a difference in the prognostic significance of sex on mortality in patients with AF [3]. The odd ratios for death in women and men with AF compared with non-AF controls were 1.9 and 1.5, respectively [3]. A recent meta-analysis reported that AF is a stronger risk factor for cardiovascular disease and death in women than in men [4]. Additionally, a study from Taiwan reported that women with AF with a CHA_2_DS_2_-VASc score of 1 demonstrated a higher risk of ischemic stroke than patients without AF, whereas no such association was observed in men with AF [29]. In our study, we noted that women showed a higher SMR than men across all age groups. Furthermore, women with AF demonstrated higher mortality rates and SMRs with respect to cerebrovascular disease and ischemic heart disease than men. The increased risk of mortality in women is attributable to several factors; although women demonstrate a higher risk of stroke, they are less likely to be prescribed OACs than men [33]. Recently, a nationwide population-based study reported that women were more likely to be undertreated with OACs than men [34]. Furthermore, women showed a higher risk of bleeding events with OAC use [35].

We observed that cardiovascular disease was the leading cause of death in patients with AF, and that these patients had a 5.7-fold higher mortality risk due to cardiovascular disease than the general population. Our results were in agreement with those of previous studies showing that cardiovascular disease was the leading cause of death in patients with AF [22, 31, 36–39]. A cohort study based on a registry of patients with AF across 47 countries revealed that heart failure was the most common cause of death, followed by stroke [9]. Another study revealed that ischemic heart disease was the most common underlying cause of death among decedents with AF [8]. A Swedish study also showed that ischemic heart disease and myocardial infarction were more common causes of death than cerebral infarction or bleeding [5]. In contrast to Western studies, we observed that cerebral infarction was the most common specific cause of cardiovascular death. Among Asians, cerebral infarction was a more common cause of death than ischemic heart disease [40–42]. East Asian countries have shown higher mortalities due to stroke and lower coronary heart disease [41]. Another large Chinese cohort study has also shown that the age-adjusted stroke mortality rate was 3-fold higher than the coronary heart disease mortality rate [42]. Moreover, in a comparative study between Asian-Americans and non-Hispanic whites, a higher cerebrovascular disease-related mortality rate was observed in Asian-Americans, whereas higher heart failure- and ischemic heart disease-related mortality rates were observed in non-Hispanic whites [43]. A possible explanation for this racial difference in the cause of death is that the slope of the relationship between blood pressure and stroke was steeper in Asians [44]. Another possible explanation is that OACs are underutilized in Asia compared with Western countries. The GARFIELD-AF registry showed that the use of OAC therapy was lower in Asia than in other regions [45]. In the GLORIA-AF phase I study, the proportion of Chinese patients with AF who take vitamin K antagonists was 20.3%. This proportion was similar to that observed in this study, but was much lower than that reported in Europe (64%) and the Middle East (45%) [46]. Underutilization of OACs was associated with an increased risk of stroke, which is generally higher in Asians than in non-Asians [47].

This study has some limitations. First, the definitions of AF and other comorbidities were based on the NHIS claims data, and the causes of death were based on ICD-10 diagnosis codes. Thus, the possibility of underestimation or overestimation of the number of patients with AF and the cause of death cannot be ignored. Nevertheless, we used the same definition and validated it in our previous studies [2, 48]. Second, although it would be reasonable to adjust several clinical variables that could affect survival, the Korean mortality database provided by Statistics Korea (a government agency) did not contain these data. In addition, detailed information about the type of AF could not be verified in these claims data. Third, this study included only Koreans; thus, our results may not be generalizable to other populations. Nevertheless, this is the largest study that included patients with AF across all age groups based on a nationwide population-based cohort, which allowed a comprehensive analysis of mortality and the assessment of detailed mortality patterns in patients with AF.

## Conclusion

In this nationwide population-based study, patients with AF showed a 4-fold higher risk of mortality than the general population. Although the absolute mortality rate was higher in older patients and men, the prognostic impact of AF was greater in younger patients and women. Diseases of the circulatory system were observed to be the leading cause of death. Notably, cerebral infarction was the most common specific disease associated with mortality, in contrast to findings in the Western population. It is important to characterize the mortality patterns to establish more effective treatment strategies for patients with AF. Therefore, optimal management of cardiovascular risk factors and close attention to preventing cerebral infarction are warranted to reduce the mortality rate in Asians with AF.

## Supporting information

S1 Fig Proportion of specific causes of death in patients with atrial fibrillation according to ICD-10 codes. Bar graphs showed causes of death in overall AF patient, men, and women.
(TIF)

S2 Fig Cause of death in atrial fibrillation patients according to age group. Number of death was abruptly increased after age of 60. Malignant neoplasms and diseases of the circulatory system were the two most common causes of death in all groups. After age of 60, Number of death due to diseases of the circulatory system exceeds the number of death due to malignancy.
(TIF)

S1 Table International Classification of Diseases (ICD) codes used in this study. (DOCX)

S2 Table Baseline characteristics of patients with atrial fibrillation in National Health Insurance Service–National Sample Cohort database. (DOCX)

S3 Table Major specific causes of death in patients with atrial fibrillation according to sex (ICD-10 code). (DOCX)
