# Supplementary figures and images for: Mortality and causes of death in patients with atrial fibrillation: A nationwide population-based study
Source: PLoS One. 2018 Dec 26;13(12):e0209687. doi: 10.1371/journal.pone.0209687 (PMC6306259; doi:10.1371/journal.pone.0209687)

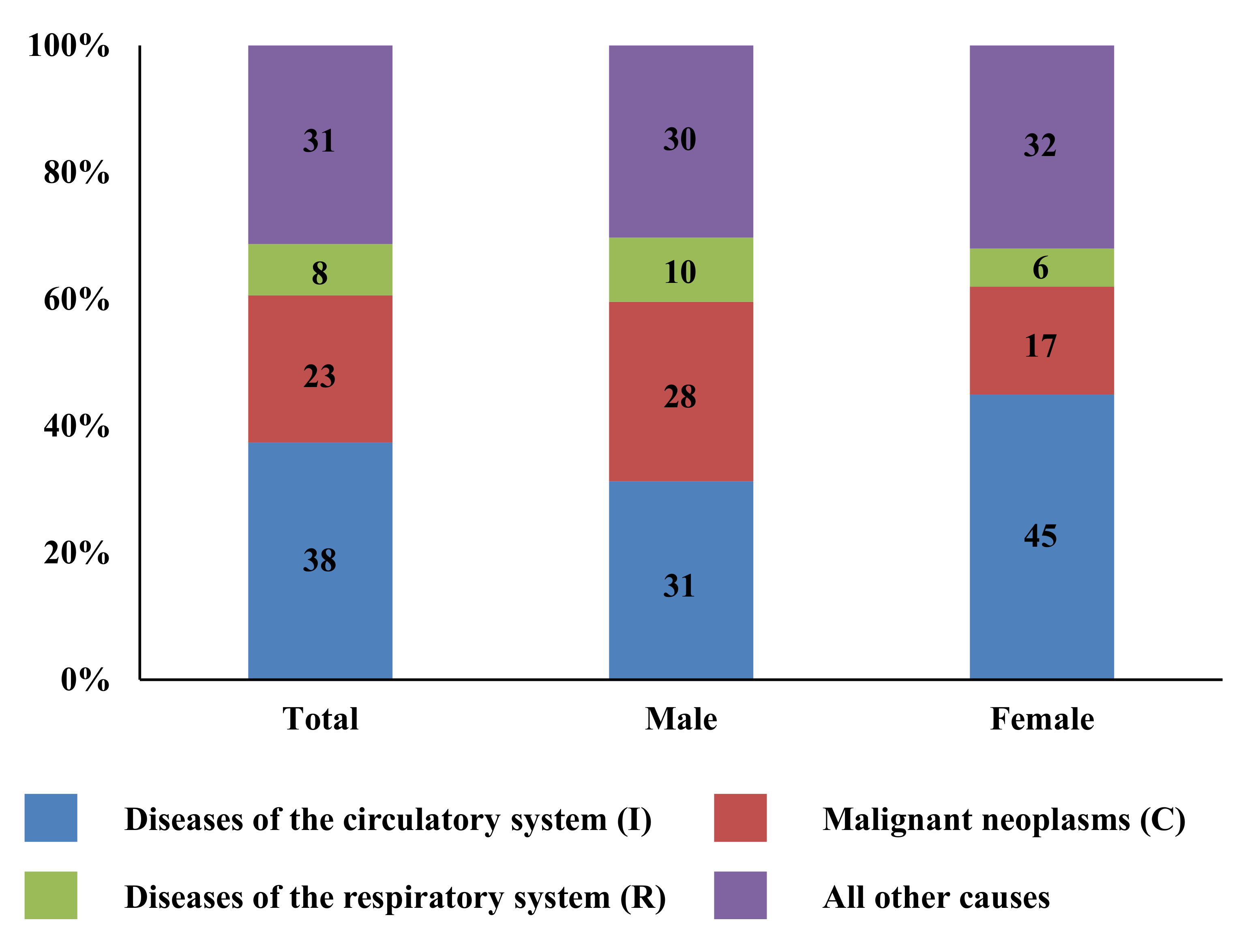

Supplement: S1 Fig — Bar graphs showed causes of death in overall AF patient, men, and women. (TIF) [file pone.0209687.s001.tif]
