# Supplementary material for: Mortality and causes of death in patients with atrial fibrillation: A nationwide population-based study
Source: PLoS One. 2018 Dec 26;13(12):e0209687. doi: 10.1371/journal.pone.0209687 (PMC6306259; doi:10.1371/journal.pone.0209687)
Supplement: S1 Table — (DOCX) [file pone.0209687.s003.docx]

**S1 Table. International Classification of Diseases (ICD) codes used in this study for defining atrial fibrillation and patients’ baseline comorbidities.**

| **Variables** | **ICD-10 codes** | **Diagnostic definition** |
| --- | --- | --- |
| **Atrial fibrillation** | Inclusion: I48  Exclusion: I50, I52, I59, Z952-Z954 | ≥1 admission or ≥2 outpatient department visits in a year |
| **Hypertension^*^** | I10-I15 | ≥1 admission or ≥2 outpatient department visits AND  Use of ≥1 anti-hypertensive medication (thiazide, loop diuretics, aldosterone antagonist, alpha blocker, beta blocker, calcium channel blocker, angiotensin converting enzyme inhibitor, and angiotensin II receptor blocker) |
| **Diabetes mellitus^*^** | E11-E14 | ≥1 admission or ≥2 outpatient department visits AND  Use of anti-diabetic medication (sulfonyurea, metformin, meglitinide, thiazolidinedione, dipeptidyl peptidase-4 inhibitor, alpha-glucosidase inhibitor, and insulin) |
| **Congestive heart failure** | I50 | ≥1 admission or ≥1 outpatient department visit |
| **Ischemic heart disease** | I20-I25 | ≥1 admission or ≥2 outpatient department visits |
| **Myocardial infarction** | I21, I22 | ≥1 admission or ≥1 outpatient department visit |
| **Peripheral artery disease** | I70, I73 | ≥1 admission or ≥1 outpatient department visits |
| **Stroke** | I63, I64 | ≥1 admission or ≥1 outpatient department visits |
| **Transient ischemic attack** | G458, G4599 | ≥1 admission or ≥1 outpatient department visits |
| **Thromboembolism** | I74 | ≥1 admission or ≥1 outpatient department visits |
| **Dyslipidemia** | E78 | ≥1 admission or ≥1 outpatient department visit |
| **Chronic lung disease** | J41-J44 | ≥1 admission or ≥1 outpatient department visit |
| **End-stage renal disease** | N18, N19, Z49, Z905, Z94, Z992 | ≥2 dialysis (hemodialysis or peritoneal dialysis) |
| **Cancer** | Any C | ≥1 admission or ≥1 outpatient department visit |

^*^ Hypertension and diabetes mellitus were identified to have the diagnosis when patients had ≥1 admission or ≥2 outpatient department visits and relevant prescription record for preventing overestimation of diagnosis.
