# Supplementary material for: Mortality and causes of death in patients with atrial fibrillation: A nationwide population-based study
Source: PLoS One. 2018 Dec 26;13(12):e0209687. doi: 10.1371/journal.pone.0209687 (PMC6306259; doi:10.1371/journal.pone.0209687)
Supplement: S2 Table — (DOCX) [file pone.0209687.s004.docx]

**S2 Table. Baseline characteristics of patients with atrial fibrillation in NHIS-NSC database.**

|  | **Total**  **(N=15,411)** | **Men**  **(N=8,226)** | **Women**  **(N=7,185)** |
| --- | --- | --- | --- |
| **Age, years** | 63.9±15.9 | 61.9±15.7 | 66.2±15.9 |
| **<20** | 215 (1.4) | 136 (1.7) | 79 (1.1) |
| **20-29** | 348 (2.3) | 192 (2.3) | 156 (2.2) |
| **30-39** | 669 (4.3) | 385 (4.7) | 284 (4.0) |
| **40-49** | 1,372 (8.9) | 873 (10.6) | 499 (6.9) |
| **50-59** | 2,515 (16.3) | 1,567 (19.0) | 948 (13.2) |
| **60-69** | 3,875 (25.1) | 2,201 (26.8) | 1,674 (23.2) |
| **70-79** | 4,118 (26.7) | 2,014 (24.5) | 2,104 (29.3) |
| **≥80** | 2,299 (14.9) | 858 (10.4) | 1,441 (20.1) |
| **Body mass index (kg/m^2^)^*^** | 24.24±3.39 | 24.15±3.24 | 24.36±3.58 |
| **Smoking status^*^** |  |  |  |
| **Non-smoker** | 4,179 (67.4) | 1,699 (47.3) | 2480 (94.9) |
| **Ex-smoker** | 879 (14.2) | 840 (23.4) | 39 (1.5) |
| **Current smoker** | 1,145 (18.5) | 1,051 (29.3) | 94 (3.6) |
| **CHA_2_DS_2_-VASc score** | 2.9±1.9 | 2.3±1.7 | 3.7±1.9 |
| **Hypertension** | 9,893 (64.2) | 5,141 (62.5) | 4,752 (66.1) |
| **Diabetes mellitus** | 3,370 (21.9) | 1,795 (21.8) | 1,575 (21.9) |
| **Congestive heart failure** | 3,674 (23.8) | 1,748 (21.3) | 1,926 (26.8) |
| **Ischemic heart disease** | 5,363 (34.8) | 2,883 (35.1) | 2,480 (34.5) |
| **Myocardial infarction** | 1,353 (8.8) | 771 (9.4) | 582 (8.1) |
| **Peripheral artery disease** | 1,820 (11.8) | 957 (11.6) | 863 (12.0) |
| **Stroke or TIA** | 2,384 (15.5) | 1,217 (14.8) | 1,167 (16.2) |
| **Dyslipidemia** | 6,482 (42.1) | 3,341 (40.6) | 3,141 (43.7) |
| **Chronic lung disease** | 3,476 (22.6) | 1,979 (24.1) | 1,497 (20.8) |
| **End-stage renal disease** | 220 (1.4) | 128 (1.6) | 92 (1.3) |
| **Cancer** | 2,173 (14.1) | 1,372(16.7) | 801 (11.2) |
| **Oral anticoagulant** | 2,759 (17.9) | 1,565 (19.0) | 1,194 (16.6) |
| **Antiarrhythmic drugs (Ic + III)** | 1,769 (11.5) | 1,068 (13.0) | 701 (9.8) |

Data are expressed as the mean ± standard deviation or as the number (percentage).

^*^Only 6,203 patients (3,590 men and 2,613 women) had information of body weight, height and smoking status.

AF, atrial fibrillation; NHIS-NSC, National Health Insurance Service-National Sample Cohort; TIA, transient ischemic attack
