# Supplementary material for: Mortality and causes of death in patients with atrial fibrillation: A nationwide population-based study
Source: PLoS One. 2018 Dec 26;13(12):e0209687. doi: 10.1371/journal.pone.0209687 (PMC6306259; doi:10.1371/journal.pone.0209687)
Supplement: S3 Table — (DOCX) [file pone.0209687.s005.docx]

**S3 Table. Major specific causes of death in patients with atrial fibrillation according to sex (ICD-10 code).**

| **Rank** | **Total** | |  | **Men** | |  | **Women** | |
| --- | --- | --- | --- | --- | --- | --- | --- | --- |
|  | **Cause of death** | **N (%)** |  | **Cause of death** | **N (%)** |  | **Cause of death** | **N (%)** |
| **1** | Cerebral infarction (I63) | 345 (7.8) |  | Malignant neoplasm of bronchus and lung (C34) | 216 (8.9) |  | Cerebral infarction (I63) | 194 (9.5) |
| **2** | Malignant neoplasm of bronchus and lung (C34) | 266 (6.0) |  | Cerebral infarction (I63) | 152 (6.3) |  | Senility (R54) | 135 (6.6) |
| **3** | Acute myocardial infarction (I21) | 221 (5.0) |  | Acute myocardial infarction (I21) | 117 (4.8) |  | Sequelae of cerebrovascular disease (I69) | 109 (5.3) |
| **4** | Senility (R54) | 200 (4.5) |  | Malignant neoplasm of liver and intrahepatic bile ducts (C22) | 99 (4.1) |  | Acute myocardial infarction (I21) | 104 (5.1) |
| **5** | Sequelae of cerebrovascular disease (I69) | 198 (4.5) |  | Chronic obstructive pulmonary disease (J44) | 90 (3.7) |  | Heart failure (I50) | 86 (4.2) |
| **6** | Type 2 diabetes mellitus (E11) | 157 (3.6) |  | Gastric cancer (C16) | 89 (3.7) |  | Type 2 diabetes mellitus (E11) | 84 (4.1) |
| 7 | Heart failure (I50) | 145 (3.3) |  | Sequelae of cerebrovascular disease (I69) | 89 (3.7) |  | Hypertensive heart disease (I11) | 72 (3.5) |
| 8 | Malignant neoplasm of liver and intrahepatic bile ducts (C22) | 139 (3.2) |  | Pneumonia, organism unspecified (J18) | 77 (3.2) |  | Atrial fibrillation and flutter (I48) | 61 (3.0) |
| 9 | Pneumonia, organism unspecified (J18) | 122 (2.8) |  | Type 2 diabetes mellitus (E11) | 73 (3.0) |  | Malignant neoplasm of bronchus and lung (C34) | 50 (2.4) |
| 10 | Malignant neoplasm of stomach (C16) | 119 (2.7) |  | Senility (R54) | 65 (2.7) |  | Chronic kidney disease (N18) | 48 (2.3) |
